# Fe65 Suppresses Breast Cancer Cell Migration and Invasion through Tip60 Mediated Cortactin Acetylation

**DOI:** 10.1038/srep11529

**Published:** 2015-07-13

**Authors:** Yuefeng Sun, Jianwei Sun, Panida Lungchukiet, Waise Quarni, Shengyu Yang, Xiaohong Zhang, Wenlong Bai

**Affiliations:** 1 Departments of Pathology and Cell Biology, University of South Florida Morsani College of Medicine, Tampa, FL 33612; 2 Department of Oncological Sciences, University of South Florida Morsani College of Medicine, Tampa, FL 33612; 3 Program of Cancer Biology and Evolution, H. Lee Moffitt Cancer Center and Research Institute, Tampa, FL 33612; 4 Comprehensive Melanoma Research Center and Department of Tumor Biology, H. Lee Moffitt Cancer Center and Research Institute, Tampa, FL 33612

## Abstract

Fe65 is a brain-enriched adaptor protein known for its role in the action of the Aβ amyloid precursor protein in neuronal cells and Alzheimer’s disease, but little is known about its functions in cancer cells. The present study documents for the first time a role of Fe65 in suppressing breast cancer cell migration and invasion. Mechanistic studies suggest that the suppression is mediated through its phosphotyrosine binding domain 1 that mediates the recruitment of Tip60 to cortactin to stimulate its acetylation. The studies identify the Tip60 acetyltransferase as a cytoplasmic drug target for the therapeutic intervention of metastatic breast cancers.

Breast cancer is the second leading cause of cancer death among women in the U.S and most of the deaths are caused by metastasis, a complex behavior of cancer cells involving migration, invasion and microenvironment remodeling^1^,^2^. The treatment for breast cancer patients with metastatic disease has made little improvement during the past 30 years^1^,^3^. Understanding the molecular mechanisms underlying breast cancer metastasis is critical for the development of new therapeutic methods.

Histone acetyltransferases (HATs) and histone deacetylases (HDACs) regulate post-translational modifications by adding or removing acetyl-groups from lysine residues of histone and non-histone proteins^4^,^5^,^6^. They regulate essentially all cellular processes including cell motility and invasion. Among all the known HATs, Tip60, a member of the MYST family, is part of an evolutionarily conserved multisubunit complex, NuA4, which is recruited by many transcription factors, including p53 and nuclear receptors^7^,^8^, to their target promoters, where it participates in essential functions such as histone acetylation, transcriptional activation, DNA repair and maintenance of stem cell function etc.^9^,^10^,^11^. A role of Tip60 in suppressing tumor invasion has been suggested by the finding that it stimulates the expression of metastatic tumor suppressor KAI1^12^ and that it is a haplo-insufficient tumor suppressor of which the expression is decreased during breast cancer development and progression^13^. Opposite to Tip60, HDAC6, a class II HDAC that is mainly localized to the cytoplasm, has been documented in the literature as a promoter of cell motility by functioning as a deacetylase for tubulin and cortactin^14^,^15^,^16^. Consistently, HDAC6 has been shown to be overexpressed in multiple cancers and cancer cell lines^17^.

Fe65 is a neuronal adaptor that has been implicated in the pathogenesis of the Alzheimer’s disease due to its binding to the carboxyl terminus of the Aβ amyloid precursor protein (APP)^18^,^19^. It contains an undefined N-terminus, a group II tryptophan-tryptophan (WW) domain in the middle and two consecutive protein tyrosine binding (PTB) domains, namely PTB1 and PTB2 at the carboxyl terminus^20^. To date, more than 20 Fe65-interacting proteins have been identified^18^. Through PTB2, Fe65 forms a multimeric complex with APP to stimulate transcription through the recruitment of CP2/LSF/LBP1 and the histone acetyltransferase Tip60^19^,^21^,^22^ to the PTB1 and assembly factor SET to the WW domain^23^. The PTB1 domain of Fe65 also interacts with two cell surface lipoproteins receptors, the low-density lipoprotein receptor related protein^24^ and ApoEr2^25^, which establishes a biological linkage between APP and the lipoprotein receptors by forming trimeric complexes with APP. A previous report has also described the WW domain as the binding site for Mena^26^, through which Fe65 may regulate the actin cytoskeleton, cell motility, and neuronal growth cone formation^27^,^28^.

Because of its importance in Alzheimer’s disease, studies in the past have mainly focused on the functions of Fe65 in neuronal cells and have shown that Fe65 plays an important role in neurogenesis^29^,^30^, neuronal migration and positioning^27^,^31^, neurite outgrowth^28^,^32^, synapse formation and learning^33^,^34^,^35^,^36^,^37^. Little is known about its functions in non-neuronal cells except that Fe65 has been implicated in DNA repair and apoptosis^38^,^39^,^40^. Essentially, nothing is known about the role of Fe65 in cancer cell migration and invasion, cellular processes essential for tumor metastasis. Published studies have linked estrogen actions to APP signaling in neuronal cells through Fe65^41^. More recent studies have defined Fe65 as a transcriptional cofactor for the estrogen receptor alpha (ERα) that potentiates estrogen stimulation of breast cancer cell growth^42^. The present studies report for the first time a role of Fe65 in suppressing breast cancer migration and invasion by showing that Fe65 binds to cortactin in ERα negative breast cancer cells and promotes its acetylation through the Tip60 acetyltransferase.

## Results

### Fe65 knockdown promotes the migration and invasion of ERα negative breast cancer cells

**Figure 1:**
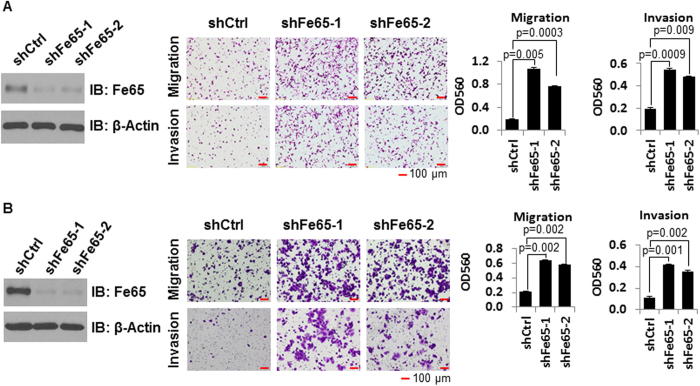
Fe65 inhibits cell motility in breast cancer cells. **(A),** 5 × 10^4^ control or Fe65 stable knockdown MDA-MB-231 cells were plated into trans-well inserts and 3% FBS was used in the medium at the bottom of 24-well plates to induce the migration and invasion. Cells were fixed and photographed 8 h after plating. **(B),** 5 × 10^4^ control or Fe65 stable knockdown MDA-MB-361 cells were plated into trans-well inserts and 5% FBS was used in the medium at the bottom of 24-well plates to induce the migration and invasion. Cells were fixed and photographed 16 h after plating. Western blots show efficient Fe65 knockdown and β-actin blots are included as loading controls. Quantitative data are shown as bar graphs with each data point representing analyses in duplicate performed in parallel that were repeated three times (n = 6). Values are means ± SD. P-values were calculated with student’s *t* test (1 tailed distribution).

In previous studies, it was noted that Fe65 was expressed at high levels in the cytoplasm of invasive breast cancer cells such as MDA-MB-231 and MDA-MB-361^42^, suggesting a possible role of Fe65 in controlling breast cancer invasion. To test this, Fe65 stable knockdown clones were established and the effect of Fe65 knockdown on cell migration and invasion assessed in trans-well assays. As shown in Fig. 1, Western blot analyses showed that Fe65 shRNA efficiently decreased Fe65 protein in the stable clones (left panels) (full blots available in the Supplementary Information). Compared to control clones, Fe65 knockdown clones exhibited increased migration and invasion in both cell lines (middle panels). Quantitative analyses showed that the increases were significant (right panels). Similar data were obtained from two independent knockdown clones, which excludes the possibility that cloning selection had contributed to the increases in migration and invasion. The above analyses suggest that Fe65 is a suppressor of breast cancer invasion and migration.

### The ability of Fe65 to suppress cell migration and invasion is sensitive to HDAC6 status

**Figure 2:**
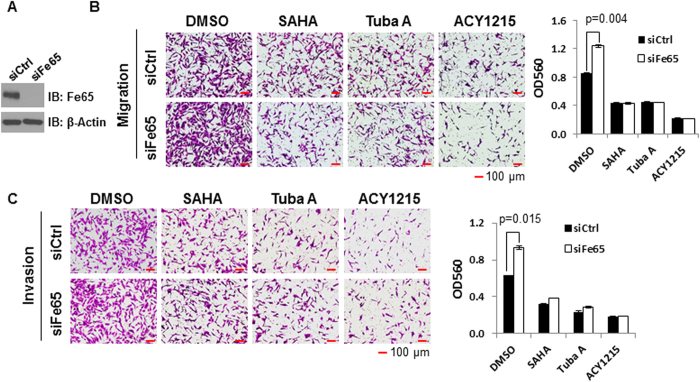
The effect of Fe65 on breast cancer cell motility is acetylation-sensitive. MDA-MB-231 cells were transfected with control or Fe65 siRNA. 40 h post transfections; cells were treated with DMSO, 1 μM SAHA, 2 μM tubastatin A (Tuba A) or 2 μg/ml ACY1215 for 8 h. **(A),** Western blot analyses were performed to show efficient Fe65 knockdown and β-actin blot was included to show even loading. **(B)** and **(C),** Cell migration (B) and invasion (C) assays were performed as in Fig. 1A and photographed 16 h after plating. Quantitative data were presented as bar graphs and data statistics performed as described in Fig. 1.

HDAC6 has been reported to control F-actin-dependent cell motility by altering the acetylation status of cortactin^15^ and Mena is an F-actin binding protein known to regulate breast cancer invasion^43^,^44^. Because Mena forms a complex with Fe65, it is thus possible that Fe65 regulates breast cancer invasion in an acetylation and HDAC6 sensitive manner. To test this, the effects of Fe65 knockdown on cell migration and invasion were examined in MDA-MB-231 cells in the presence or absence of the pan-HDAC or HDAC6-selective inhibitors. As shown in Fig. 2A, transient transfection with Fe65 siRNA significantly decreased Fe65 protein expression. Treatments with SAHA, tubastatin A or ACY1215 decreased cell migration and invasion (Fig. 2B,C, upper panels), which is consistent with the positive role of HDAC6 in cell migration and invasion documented in published studies^15^. Fe65 knockdown increased cell migration (Fig. 2B) and invasion (Fig. 2C) in vehicle treated cells but not in cells treated with HDAC inhibitors, indicating an acetylation and HDAC6-sensitive effect of Fe65 on cell migration and invasion. The increase in cell migration and invasion caused by Fe65 siRNA is smaller than what was detected in Fe65 stable knockdown clones (Fig. 1), possibly due to the effect of transfection reagents or the vehicle (DMSO) for HDAC inhibitors.

**Figure 3:**
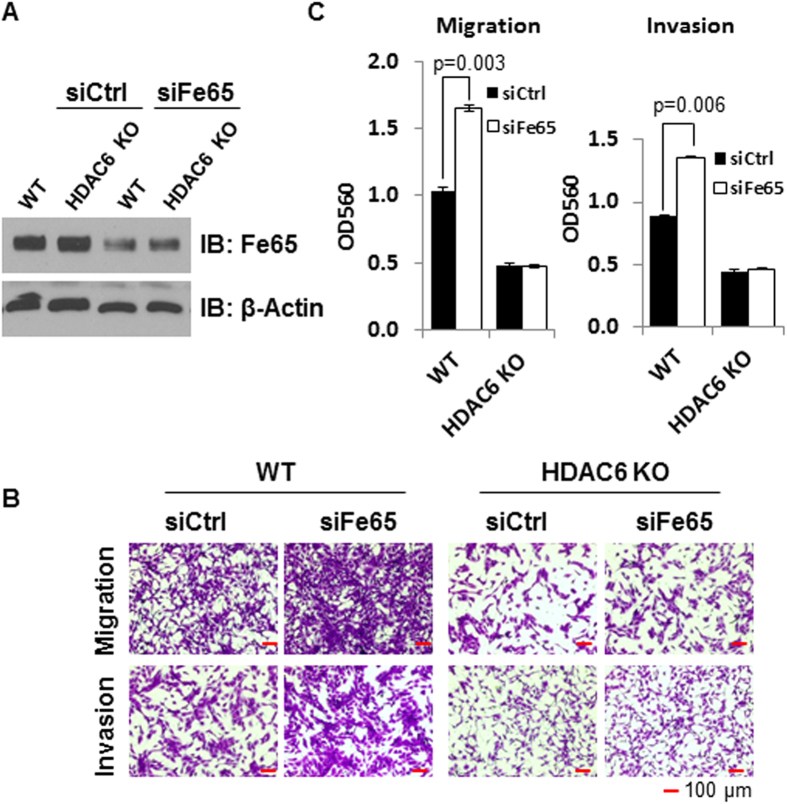
The ability of Fe65 to suppress cell motility depends on HDAC6 status. WT and HDAC6 KO MEFs were transfected with control or Fe65 siRNA for 48 h. **(A),** Western blots were performed to show the efficient knockdown of Fe65 and β-actin blot was included to show even loading. **(B),** 4 × 10^4^ cells were plated into trans-well inserts and cells were fixed and photographed 16 h after plating. **(C),** Quantitative data were presented as bar graphs and data statistics performed as described in Fig. 1.

To further validate the role of HDAC6 in the Fe65 action, we investigated the effect of Fe65 knockdown on cell motility in wild type (WT) and HDAC6 null (HDAC6 KO) mouse embryonic fibroblasts (MEFs). Although Fe65 siRNA decreased Fe65 protein expression to similar levels in WT and HDAC6 KO MEFs (Fig. 3A), it selectively increased migration and invasion of the WT, but not HDAC6 KO, MEFs (Fig. 3B). Quantitative data showed that the effect of Fe65 knockdown on the migration and invasion of WT MEFs is significant (Fig. 3C). Together with the HDAC inhibitor analyses in Fig. 2, the data from HDAC6 KO MEFs have clearly shown that the ability of Fe65 to suppress cell motility and invasion is sensitive to protein acetylation events controlled by HDAC6.

### Fe65 binds to cortactin through its PTB1 domain to suppress cell motility and invasion

**Figure 4:**
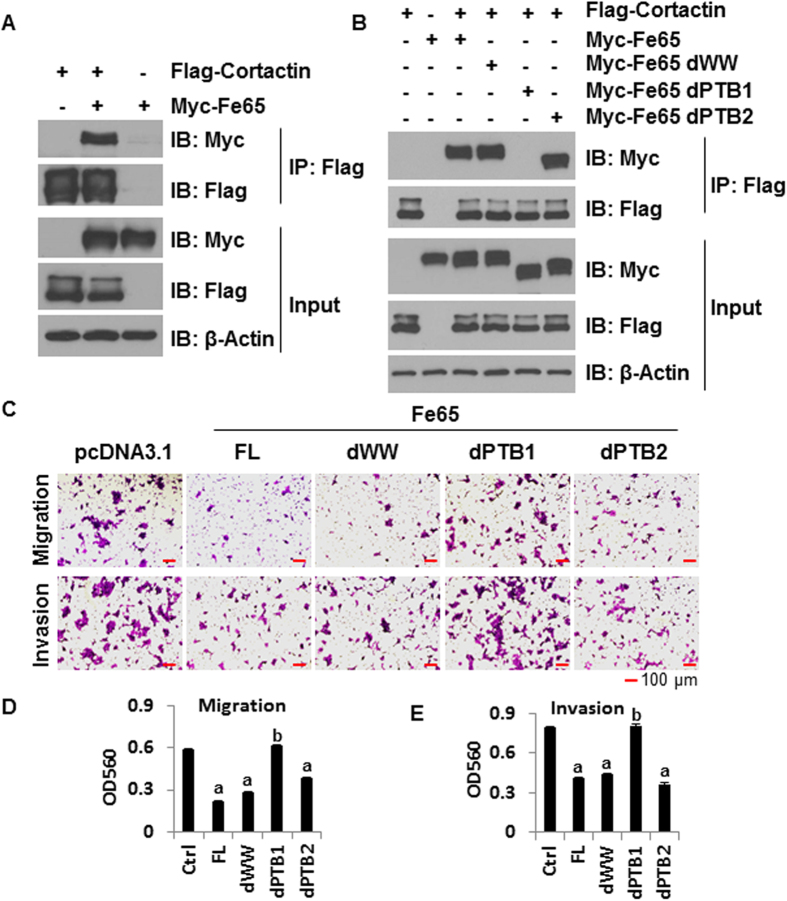
Fe65 binds to cortactin to inhibit cell motility through its PTB1 domain. **(A),** 293T cells were transfected with 1.5 μg Flag-cortactin and/or 1.5 μg of Myc-Fe65. Cellular extracts were subjected to co-immunoprecipitation analyses with antibodies as indicated. **(B),** 293T cells were transfected with 1.5 μg Flag-cortactin and 1.5 μg of Myc-Fe65 or deletion constructs as indicated. Cellular extracts were subjected to co-immunoprecipitation analyses with antibodies as indicated. **(C),** 293T cells were transfected with Myc-Fe65 or deletion constructs as indicated. 5 × 10^5^ transfected cells were plated into trans-well inserts 24 h post transfections. Cells were fixed and photographed 36 h after plating. **(D)** and **(E),** Quantitative data were presented as bar graphs and data statistics were performed as described in Fig. 1. a, p < 0.01; b, p > 0.05.

Because HDAC6 deacetylates cortactin to promote cell motility^15^, its involvement in the regulation of breast cancer cell invasion by Fe65 prompted us to investigate the possible interaction between Fe65 and cortactin. 293T cells were co-transfected with Flag-tagged cortactin and Myc-tagged Fe65 and co-immunoprecipitations performed. As shown in Fig. 4A, Flag beads co-precipitated Fe65 and cortactin in co-transfected cells. The co-precipitations reflect a true interaction between Fe65 and cortactin because the Flag beads did not precipitate Fe65 in cells transfected with Fe65 without Flag-cortactin.

To define the domain in Fe65 that mediates the interaction with cortactin, the ability of different Fe65 deletions constructs^42^ to interact with cortactin was compared in similar co-immunoprecipitation analyses. As shown in Fig. 4B, Flag beads co-precipitated with cortactin the full length (FL) Fe65 and Fe65 mutants in which the WW (dWW) or PTB2 (dPTB2) domain was deleted. Interestingly, the deletion of PTB1 (dPTB1) domain disrupted the interaction, identifying the PTB1 domain as the cortactin-binding site.

To test if the biochemical interaction mediates the biological effects of Fe65 on cell migration and invasion, different Fe65 deletion constructs were transfected into 293T cells and their effects on cell motility and invasion assessed in trans-well assays. As shown in Fig. 4C, the ectopic expression of FL Fe65, the dWW or the dPTB2 mutant significantly decreased cell migration and invasion; whereas the ectopic expression of the dPTB1 mutant had no effect on cell migration and invasion. Quantitative data showed a significant difference in cell migration and invasion between control cells and cells transfected with FL Fe65, the dWW or the dPTB2 mutant (Fig. 4D,E). Theses analyses have shown that the PTB1 domain, the cortactin-binding site, is essential for the ability of Fe65 to suppress cell migration and invasion.

### Fe65 mediates the binding of Tip60 to cortactin as well as the ability of Tip60 to acetylate cortactin and suppress cell migration and invasion

**Figure 5:**
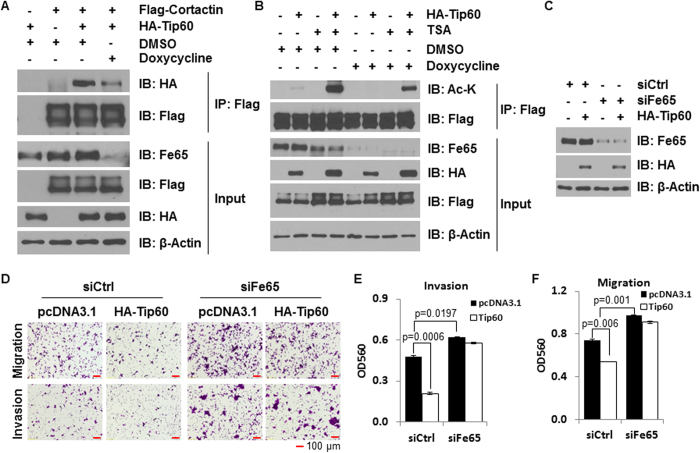
Tip60 binds to and acetylates cortactin through Fe65 to inhibit cell motility. **(A),** Inducible Fe65 stable knockdown clones of 293T cells were transfected with 1.5 μg Flag-cortactin and 1.5 μg of HA-Tip60 and treated with DMSO or 1 μg/mL doxycycline as shown. Cellular extracts were subjected to co-immunoprecipitation analyses with antibodies as indicated. **(B),** Cells were transfected as in panel A and treated with DMSO, 300 ng/mL TSA and/or 1 μg/mL doxycycline. Cellular extracts were subjected to co-immunoprecipitation analyses with antibodies as indicated. Acetyl-lysine (Ac-K) antibody is an antibody that specifically recognizes lysine-acetylated proteins. **(C)** and **(D),** 293T cells were transfected with HA-Tip60 together with control or Fe65 siRNA. 36 h post transfections, 5 × 10^5^ transfected cells were plated into the inserts for trans-well assays. Western blots were performed to show Fe65 knockdown and β-actin blots were included as loading controls (panel C). Cells were fixed and photographed 24 h after plating (panel D). **(E)** and **(F),** Quantitative data were presented as bar graphs and data statistics performed as described in Fig. 1.

As mentioned earlier, Tip60 is HAT known to interact with the PTB1 domain of Fe65^19^. It is thus reasonable to speculate that Tip60 may work through Fe65 to acetylate cortactin and suppress cell migration and invasion. To test this concept, we engineered 293T cells in which Fe65 knockdown is inducible and examined the interaction between co-transfected Flag-cortactin and HA-Tip60 in co-immunoprecipitation assays. As shown in Fig. 5A, Flag beads co-precipitated Tip60 and cortactin in co-transfected cells but not in cells transfected with HA-Tip60 or Flag-cortactin alone, showing a true binding of Tip60 to cortactin. More importantly, doxycycline treatment, which reduced the expression of Fe65 protein as expected due to induced expression of Fe65 shRNA (Fig. 5A), decreased the amount of Tip60 co-precipitated with cortactin (Fig. 5A). The data suggest that Fe65 is the adaptor that mediates the binding of Tip60 to cortactin.

To assess the effect of Tip60 on cortactin acetylation and its dependency on Fe65, the same doxycycline inducible cells were co-transfected with Flag-cortactin and HA-Tip60 and treated with vehicle or trichostatin A (TSA). The status of cortactin acetylation was assessed by immunoblotting analyses of Flag immunoprecipitates with an acetyl-lysine antibody. As shown in Fig. 5B, ectopic Tip60 expression increased the levels of cortactin acetylation in the absence of doxycycline. Doxycycline treatment, which clearly decreased the expression of endogenous Fe65, diminished the positive effect of ectopic Tip60 on cortactin acetylation. In the presence of TSA, cortactin acetylation by Tip60 was dramatically increased, which was reversed by the inducible Fe65 knockdown, but not to levels below what was induced by Tip60 in the absence of TSA and tetracycline. Together with the binding analyses, the data demonstrate that Fe65 promotes cortactin acetylation through the recruitment of Tip60 to cortactin.

The Fe65-dependent effect of Tip60 on cortactin binding and acetylation raises the possibility for Tip60 to regulate cell migration and invasion through Fe65. Indeed, ectopic Tip60 expression decreased migration and invasion of 293T cells and co-transfection of Fe65 siRNA, which effectively reduced the levels of Fe65 protein expression (Fig. 5C), blocked the ability of ectopic Tip60 to decrease cell migration and invasion (Fig. 5D,E and 5F). The data support the concept that Tip60 suppresses cell migration and invasion through Fe65-mediated complex formation with cortactin.

### Fe65 suppresses invadopodia formation in breast cancer cells

**Figure 6:**
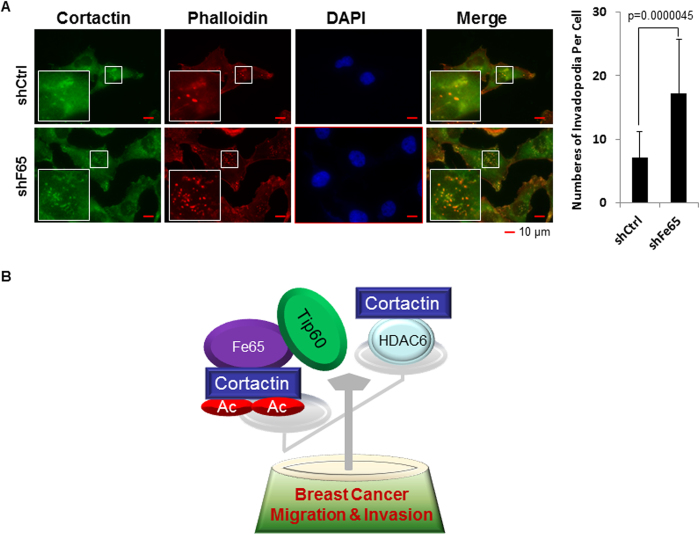
Fe65 inhibits invadopodia formation in breast cancer cells. **(A),** MDA-MB-231 cells stably expressing control or Fe65 shRNA were plated onto gelatin-coated glass coverslips and stained for cortactin (green), F-actin (phalloidin, red) and DAPI (blue). Representative immunofluorescence images were included to show invadopodia underneath the cells as orange dots in the merged panels. Quantitative data were presented as bar graphs to show the difference between control and Fe65 knockdown cells. Each data point represents the counting of 30 cells. Values are means ± SD. Statistical analyses were performed with student’s t test (1 tailed distribution). **(B)**, A model depicting our current understanding how Fe65 suppresses breast cancer migration and invasion through the recruitment of Tip60 that opposes the activity of HDAC6 on cortactin acetylation.

Invadopodia are F-actin driven adhesive membrane protrusions that coordinate extracellular matrix degradation and invasion in cancer cells^45^,^46^. The binding of Fe65 to cortactin suggests that Fe65 may regulate invadopodia formation. As shown in Fig. 6A, stable Fe65 knockdown significantly increased invadopodia formation in MDA-MB-231 cells as measured by co-immunofluorescence staining of cortactin and F-actin (with phalloidin). The data suggest a negative role of Fe65 in invadopodia formation, possibly through the increased cortactin acetylation, which is known to decrease its interaction with F-actin^15^.

## Discussion

Fe65 has been relatively well-studied for APP signaling in Alzheimer’s disease but little is known about its functions in cancer cells. It has been shown recently that Fe65 binds to the ERα and promotes estrogen-induced cell growth in breast cancer cells^42^. The present studies have extended the function of Fe65 to ERα negative breast cancers by showing for the first time that it suppresses cell migration and invasion by recruiting Tip60 to cortactin and stimulating its acetylation (Fig. 6B). Multiple lines of evidences are presented here to support the above conclusion. Firstly, Fe65 knockdown increased migration and invasion in MDA-MB-231 and MDA-MB-361 cells, identifying endogenous Fe65 as a potential suppressor of breast cancer metastasis. Secondly, Fe65 bound to cortactin through its PTB1 domain and inhibited cell migration and invasion through the same domain. Thirdly, Tip60 bound to and acetylated cortactin and suppressed cell migration and invasion, which were compromised by inducible Fe65 knockdown. Overall, the studies suggest that Fe65 may play a dual role in breast tumorigenesis. It stimulates the growth of ERα-positive tumors by serving as an ERα coactivator but suppresses metastasis of ERα-negative tumors through Tip60-mediated cortactin acetylation.

Mena is a member of Ena/VASP family of actin-binding proteins that acts as a key mediator of cytoskeletal arrangement. It enhances tumor cell migration by interfering with the activity of inhibitory capping proteins and increasing actin filament elongation rates, thereby promoting actin polymerization^44^,^47^. It is alternately spliced to give rise to multiple isoforms that are differentially expressed during tumor progression^48^. MenaINV is expressed exclusively in invasive tumor cells^48^ and Mena11a is an epithelial-specific isoform expressed in primary breast carcinomas and down-regulated in invasive tumor cells^49^. Mena∆v6, lacking the internal exon 6, is antagonistic to the role of Mena11a in cell invasion and migration in breast tumors^43^. Given the fact that Mena binds to the WW domain of Fe65^26^, it is unexpected that the deletion of WW domain did not appear to alter the ability of Fe65 to suppress cell motility and invasion (Fig. 4). Since our analyses with Fe65 truncation mutants were performed under overexpression conditions, it is important to point out that our studies do not exclude the possibility that the ability of Fe65 to regulate breast cancer invasion may be sensitive to cellular status of Mena. Similarly, the ability of Fe65 to suppress cell migration and invasion may also be subjected to regulations by APP and its C-terminal fragment after γ-secretase cleavage under *in vivo* conditions even though the PTB2 deletion appeared expendable for this new function of Fe65 under overexpression conditions in 293T cells.

Cortactin is a multi-domain protein that was first identified as a Src substrate and subsequently shown to be involved in a variety of cellular processes, including cancer progression, invasion and metastasis^50^. In invasive breast cancer cells, cortactin was shown to be present in membrane protrusions, or invadopodia, which carry proteases that digest the extracellular matrix^51^,^52^. The present study is the first to show that Fe65 binds to cortactin and increases its acetylation to suppress breast cancer migration and invasion, possibly through its negative effect on invadopodia formation. While the concept that Fe65 suppresses breast cancer invasion through cortactin binding and acetylation is novel, the signal pathways regulating the binding and acetylation as well as the mechanisms underlying the suppression of invadopodia formation remain to be defined. Cortactin phosphorylation and its functional consequences have been well studied with multiple reports documenting a correlation between high levels of tyrosine phosphorylation and elevated cell migration and cancer metastasis^50^. Src kinases are known to stimulate cortactin phosphorylation and promote invadopodia formation^53^,^54^. It will be interesting to investigate the potential crosstalk between cortactin phosphorylation mediated by Src and the acetylation mediated by Fe65-Tip60 complex in regulating invadopodia formation and breast cancer invasion and metastasis.

Many cancers display molecular alterations that disrupt the balance between HAT and HDAC activities^55^. HDACs usually exert a pro-oncogenic effect while mutations of HAT proteins have been identified in colorectal, breast, prostate, gastric and ovarian cancer^56^,^57^,^58^,^59^. Previous studies have shown that PCAF acetylates cortactin and that HDAC6 deacetylates it^15^. However, no significant difference in the levels of cortactin acetylation was detected between WT and PCAF-null MEFs^15^, suggesting the existence of additional acetyltransferases that may acetylate cortactin. The present studies identify Tip60 as a new HAT that acetylates cortactin, revealing a novel function for Tip60. Tip60 has been well studied for its nuclear activities in apoptosis, DNA damage and transcriptional regulation and its down-regulation has been found in many forms of cancer^60^. In breast cancer, Tip60 is a haplo-insufficient tumor suppressor of which the decreased transcriptional activity, protein expression or altered cellular localization was associated with tumor progression^12^,^13^. It is required for oncogene induced DNA damage response and the expression of metastasis suppressor KAI1^12^. However, the role of Tip60 is complicated as it has also been shown to deacetylate Twist, a process required for the activation of the Twist/BRD4/P-TEFb/RNA-Pol II complex, which in turn induces WNT5A expression and promotes invasion^61^. Our studies support a more direct role of Tip60 in controlling cytoskeleton remodeling and suppressing the invasive behavior of breast tumor cells, arguing that its activation may be a viable approach to deter breast tumor metastasis.

In conclusion, the present study showed for the first time that Fe65 can suppress breast cancer cell motility by promoting the acetylation of cortactin by Tip60. Targeted enhancement of the signaling through the Fe65-cortactin pathway, by either HDAC6 inhibition or Tip60 activation, may lead to the development of new therapeutic drugs that are effective for patients with metastatic breast cancers.

## Methods

### Reagents and antibodies

Collagen (C7661), TSA (T8552), anti-c-Myc antibody (C3956), anti-Flag antibody (F7425) and anti-Flag affinity gels (A2220) were purchased from Sigma-Aldrich (St. Louis, MO). Phalloidin (A12379), fetal bovine serum (FBS) (Cat, 10082-147) and lipofectamine 2000 were from Life Technologies (Grand Island, NY). Matrigel (356230) was purchased from Corning (Tewksbury, MA). Anti-hemagglutinin (anti-HA.11; PRB-101P) antibody was from Covance (Princeton, NJ). Anti-cortactin (05–180) was from EMD Millipore (Billerica, MA). Antibodies specific to Fe65 (#2877) and acetyl-lysine (#9681, Ac-K-103) were from Cell Signaling Company (Boston, MA). Anti-β-Actin (AC-15, sc-69879) was from Santa Cruz Biotechnology (Santa Cruz, CA). Fe65 (siFe65) (sequence: CUACGUAGCUCGUGAUAAG) and scrambled control (siCtrl) siRNA were obtained from Dharmacon/Thermo Scientific (Waltham, MA). The ECL Western blotting substrates were from Thermo Scientific. SAHA, tubastatin A and ACY1215 were obtained from APExBio Company (Boston, MA). All plasmids had been previously described^15^,^42^.

### Cell culture and Fe65 knockdown

293T, WT and HDAC6 KO MEFs, MDA-MB-231 and MDA-MB-361 cells were cultured in Dulbecco’s Modified Eagle’s Medium (DMEM) containing 2 mM L-glutamine, 100 units/ml penicillin, 100 μg/ml streptomycin, and 10% FBS. For the transient knockdown, cells were transfected with control or Fe65 siRNA for 48 h before migration and invasion assays. For the stable knockdown in MDA-MB-231 and MDA-MB-361, cells were transfected with the retroviral pGFP-V-RS control or Fe65 shRNA plasmids from Origene (Rockville, MD) and 24 h post transfections, were continuously cultured in DMEM medium containing 1 μg /mL puromycin. Control and Fe65 knockdown clones were isolated and Fe65 protein expression was determined by Western blot analyses. For the inducible knockdown of Fe65 in 293T, cells were transfected with pTRIPZ-control or pTRIPZ-Fe65 shRNA plasmid from GE Healthcare Bio-Sciences (Pittsburgh, PA). 24 h post transfections, stable clones were selected in media containing 1.5 μg /mL puromycin. Inducible Fe65 knockdown in isolated clones was verified by Western blot analyses in the presence or absence of 1 μg/mL doxycycline.

### Migration and invasion assays

Trans-well assays were performed as previously described^62^. Briefly, 24-well trans-well migratory cell inserts (8 μm pores, Fisher Scientific) were coated with 50 μg collagen and incubated at 4 °C overnight for migration assays. For the invasion assay, the inserts were coated with 50 μg matrigel and incubated at 37 °C for 2 h. Cells in serum-free medium were seeded into the inserts in duplicates and allowed to migrate through the inserts for different times in a 37 °C incubator. Cells that did not migrate were removed with a cotton swab and migratory cells were fixed with 4% paraformaldehyde and stained with crystal violet in ethanol. Snapshots were taken of the migratory cells. For quantification, cells were distained in 2% SDS and the absorbance was read at 560 nm.

### Immunological analyses

For immunoprecipitations, cells were suspended in lysis buffer containing 20 mM Tris-HCl (pH 7.5), 150 mM NaCl, 1 mM EDTA, 1% (v/v) NP-40, 1 mM PMSF, and protease inhibitor cocktail and sonicated for 6 seconds with two repeats. The lysates were cooled on ice for 10 minutes before centrifugation. Cellular extracts were incubated with primary antibodies conjugated with beads overnight at 4 °C. The beads were washed with cold lysis buffer for five times and boiled in SDS–PAGE sample buffer. Eluted samples were then subjected to immunoblotting analyses.

For immunoblotting analyses, precipitates or cellular extracts were separated in SDS-PAGE, transferred to a nitrocellulose membrane, and probed with cognate antibodies. Horseradish peroxidase-linked secondary antibodies and enhanced chemiluminescence reagents (Thermo Scientific, Waltham, MA) were used for protein detections.

### Invadopodia formation assays

The invadopodia formation assays were as previously described with modifications^63^,^64^. Briefly, 3 × 10^4^ cells were plated onto Texas Red-labeled and gelatin-coated glass coverslips (18 mm) and incubated for 24 h. Then, cells were fixed in 4% paraformaldehyde, permeabilized in diluting buffer (2% BSA, 0.1% Triton X-100 in PBS) and immune-stained for cortactin and F-action (with Alexa Fluor 594 phalloidin) for 30 min. Extensive washes were carried out between each step. The coverslips were then mounted onto slides and images captured using a Zeiss fluorescence microscope. To quantify invadopodia formation, 30 cells were randomly selected and counted and the average number of invadopodia per cell was calculated.

## Additional Information

**How to cite this article**: Sun, Y. *et al.* Fe65 Suppresses Breast Cancer Cell Migration and Invasion through Tip60 Mediated Cortactin Acetylation. *Sci. Rep.* **5**, 11529; doi: 10.1038/srep11529 (2015).

## Supplementary Material

Supplementary Information
