# Supplementary material for: Fe65 Suppresses Breast Cancer Cell Migration and Invasion through Tip60 Mediated Cortactin Acetylation
Source: Sci Rep. 2015 Jul 13;5:11529. doi: 10.1038/srep11529 (PMC4499803; doi:10.1038/srep11529)
Supplement: Supplementary Information [file srep11529-s1.pdf]

**Supplementary Information**

# Fe65 Suppresses Breast Cancer Cell Migration and Invasion through Tip60 Mediated Cortactin Acetylation

Yuefeng Sun[1], Jianwei Sun[4], Panida Lungchukiet[1], Waise Quarni[1], Shengyu Yang[4], Xiaohong Zhang[1, 2, 3] and Wenlong Bai[1, 2, 3, *]

**Fig. S1. Level s of Fe65 and HDAC6 protein expression in different cell lines.** Wild type (WT) and HDAC6 knockout (KO) MEFs, 293T, MDA-MB-231, MDA-MB-361 cells were lysed and cellular extracts subjected to immunoblotting analyses with indicated antibodies. HDAC6(H): anti-human HDAC6 antibody ; HDAC6(M): anti-mouse HDAC6 antibody.

The uncropped images with molecular weight markers of all Western blot data in the manuscript are presented in the supplementary information

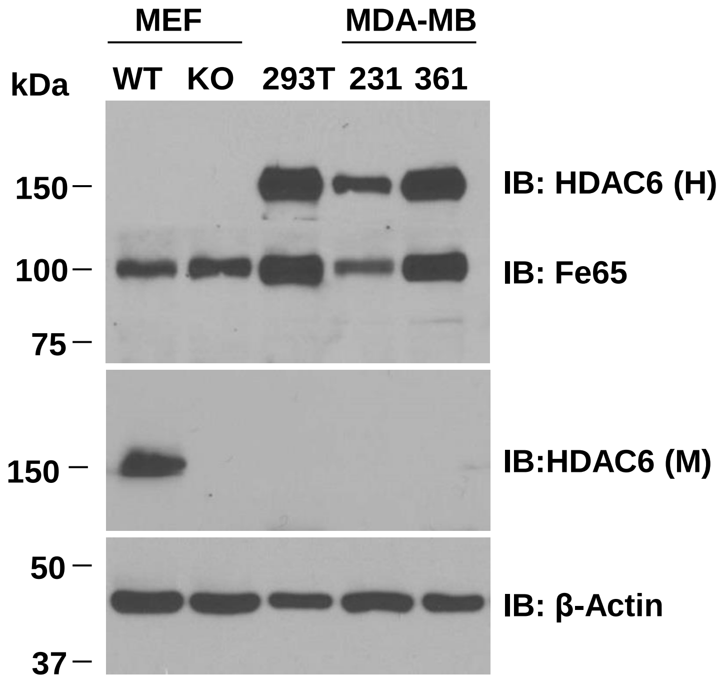


Fig S1

**Fig. S1: Level s of Fe65 and HDAC6 protein expression in different cell lines.**

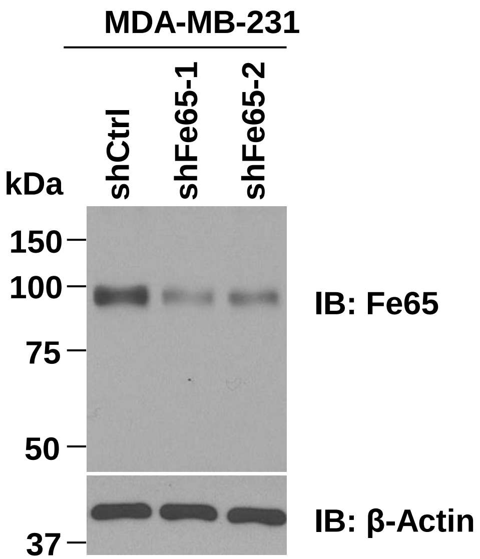


Fig. 1a

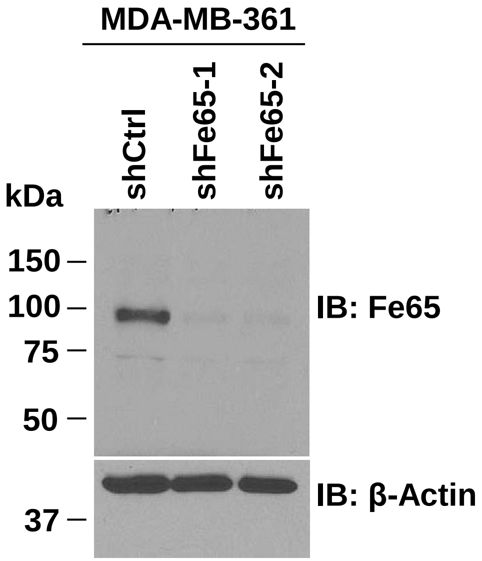


Fig. 1b

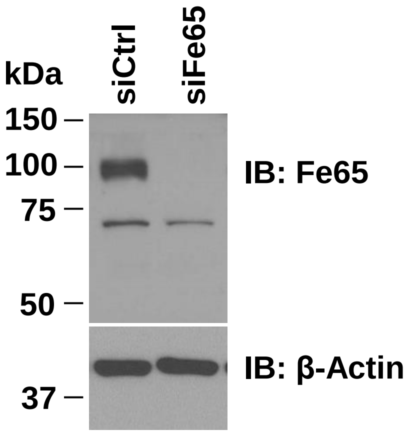


Fig. 2a

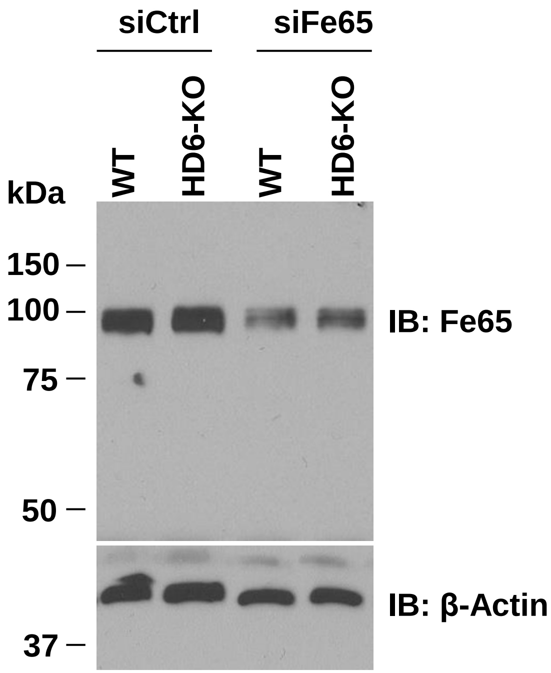
siCtrl
siFe65
WT
HD6-KO
WT
HD6-KO
kDa
150
100
75
50
37
IB: Fe65
IB: β-Actin

Fig. 3a

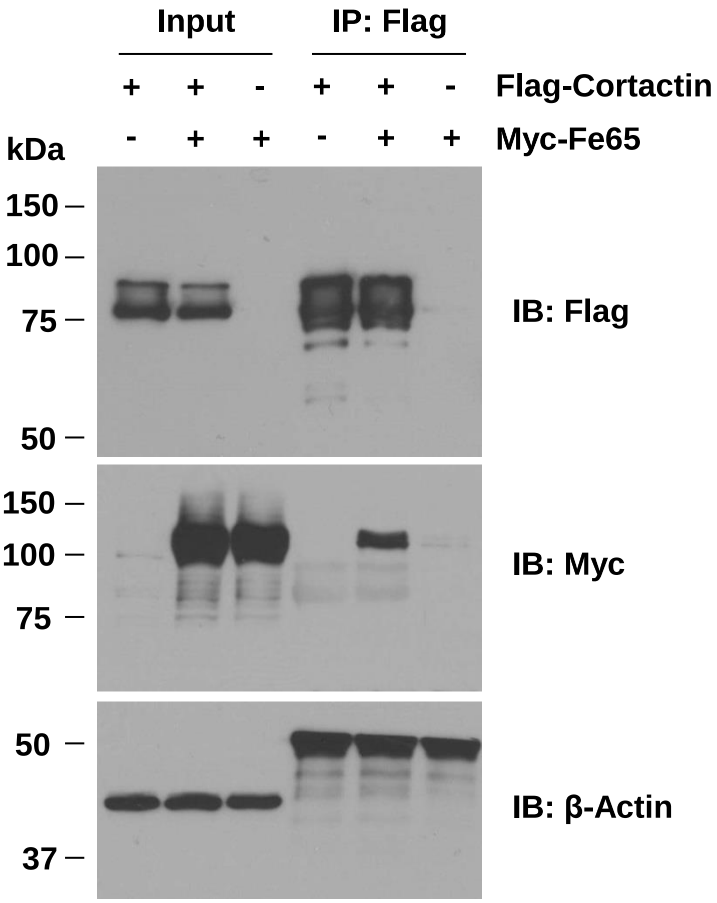
Input
IP: Flag
+ + - + + - Flag-Cortactin
- + + - + + Myc-Fe65
kDa
150
100
75
50
IB: Flag
150
100
75
IB: Myc
50
37
IB: β-Actin

Fig. 4a

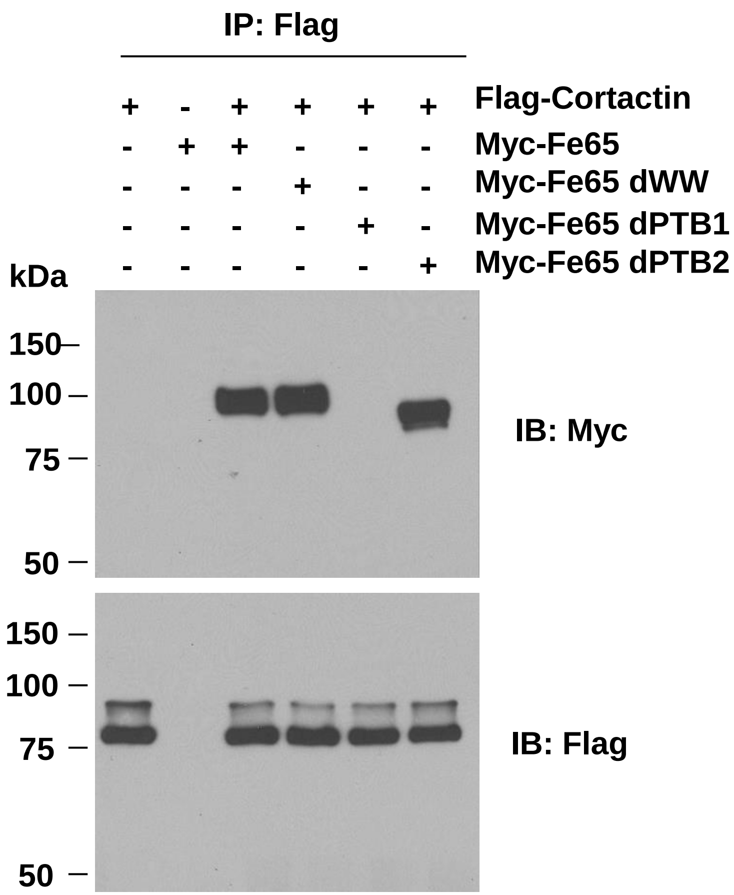
IP: Flag
Flag-Cortactin + - + + + +
Myc-Fe65 - + + - - -
Myc-Fe65 dWW - - - + - -
Myc-Fe65 dPTB1 - - - - + -
Myc-Fe65 dPTB2 - - - - - +
kDa
150
100
75
50
IB: Myc
150
100
75
50
IB: Flag

Fig. 4b

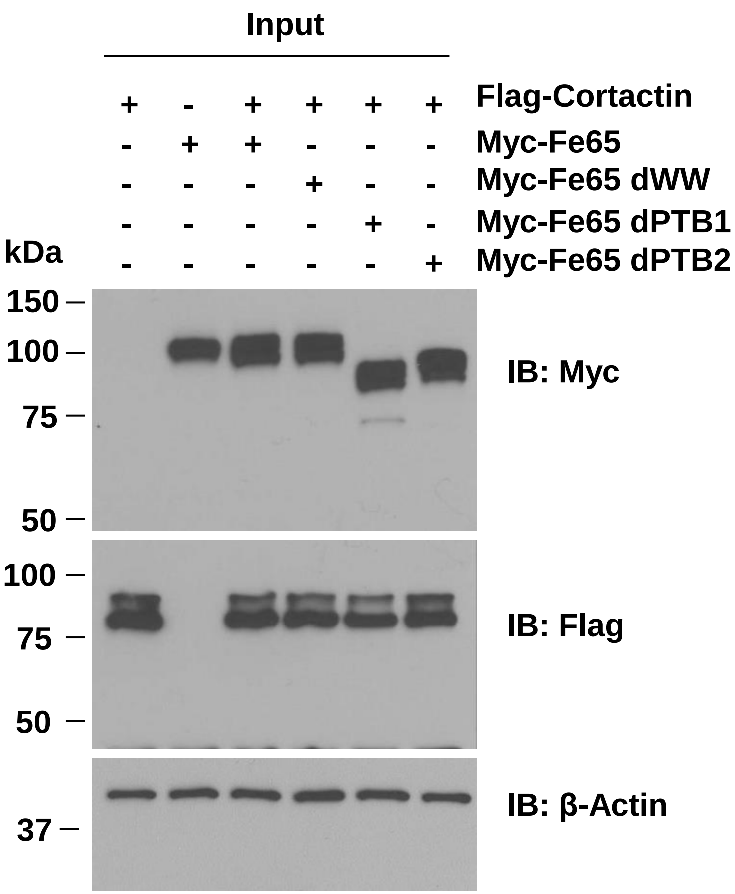
Input
Flag-Cortactin + - + + + +
Myc-Fe65 - + + - - -
Myc-Fe65 dWW - - - + - -
Myc-Fe65 dPTB1 - - - - + -
Myc-Fe65 dPTB2 - - - - - +
kDa
150
100
75
50
IB: Myc
100
75
50
IB: Flag
37
IB: β-Actin

Fig. 4b

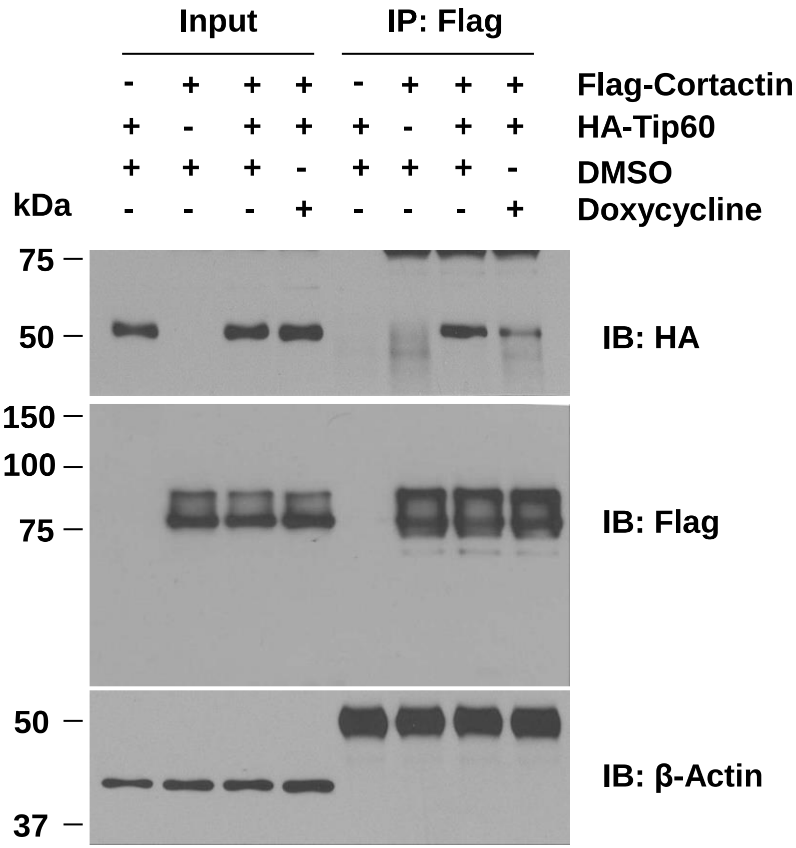
Input
IP: Flag
Flag-Cortactin
HA-Tip60
DMSO
Doxycycline
kDa
75
50
150
100
75
50
37
IB: HA
IB: Flag
IB: β-Actin

Fig. 5a

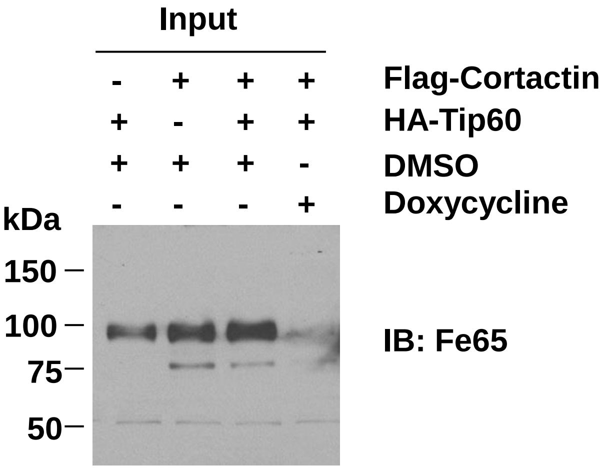
Input
Flag-Cortactin
HA-Tip60
DMSO
Doxycycline
kDa
150
100
75
50
IB: Fe65

Fig. 5a

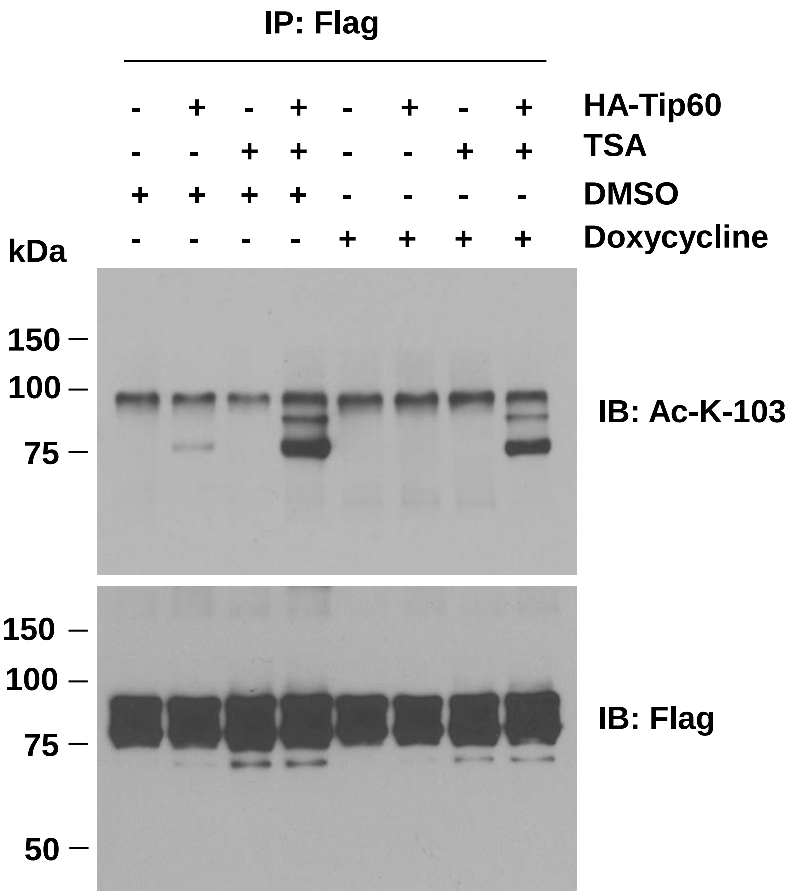
IP: Flag
HA-Tip60
TSA
DMSO
Doxycycline
kDa
150
100
75
50
IB: Ac-K-103
IB: Flag

Fig. 5b

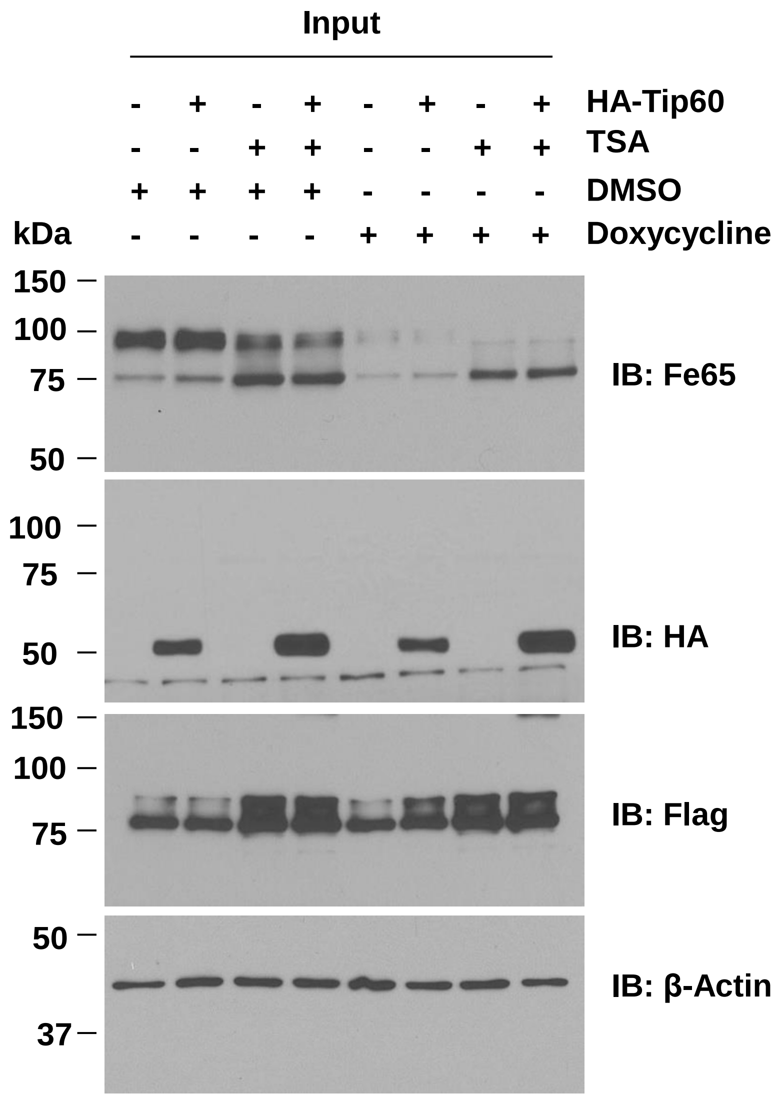
Input
HA-Tip60
TSA
DMSO
Doxycycline
kDa
150
100
75
50
37
IB: Fe65
IB: HA
IB: Flag
IB: β-Actin

Fig. 5b

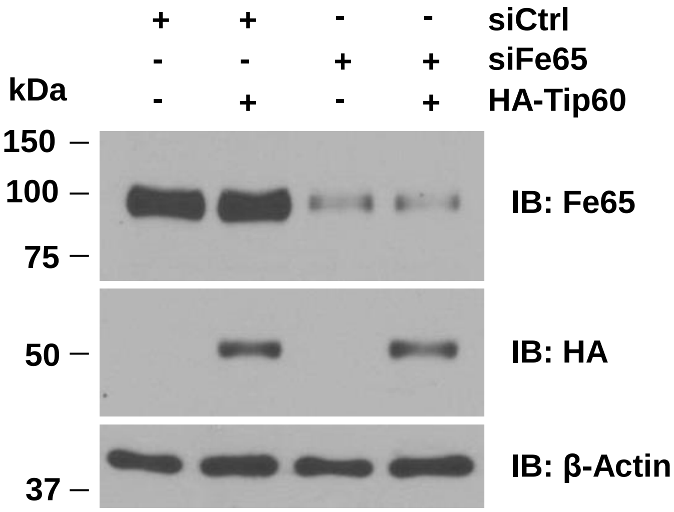
+ + - - siCtrl
- - + + siFe65
kDa
- + - + HA-Tip60
150
100
75
IB: Fe65
50
IB: HA
37
IB: β-Actin

Fig. 5c
